# User perceptions of machine learning models as decision support for colorectal cancer multidisciplinary team conferences (AID-SIM-2): a qualitative simulation study

**DOI:** 10.2340/1651-226X.2026.45754

**Published:** 2026-07-31

**Authors:** Karoline Bendix Bräuner, Birgitte Bruun, Claus Anders Bertelsen, Vegar Johansen Dagenborg, Kristina Safir-Hansen, Rasmus Sanko, Ismail Gögenur, Lars Konge

**Affiliations:** a Center for Surgical Sciences, Zealand University Hospital, Køge, Denmark; b Copenhagen Academy for Medical Education and Simulation (CAMES), Copenhagen, Denmark; c Copenhagen University Hospital – North Zealand, Hillerød, Denmark; d Department of Clinical Medicine, Faculty of Health and Medical Sciences, University of Copenhagen, Copenhagen, Denmark; e Department of Surgical Oncology, The Norwegian Radium Hospital, Oslo University Hospital, Nydalen, Norway; f The Hospital of Mid and Western Zealand, Slagelse, Slagelse, Denmark; g Charlie Tango, Copenhagen, Denmark

**Keywords:** Medical simulation, machine learning, colorectal cancer prediction models, multidisciplinary team conferences

## Abstract

**Background:**

Multidisciplinary team (MDT) conferences are considered a cornerstone of decision-making in cancer diagnostics and care. However, the current literature has not demonstrated improved patient outcomes based on the decisions of the MDT conferences.

**Aim:**

We aimed to evaluate how four different decision-support modalities impacted the decision-making process and the internal discussions in the MDTs in a multicenter simulation study, with a focus on user perceptions.

**Methods:**

Four colorectal cancer centers with MDTs participated. We performed four simulations in each center. Each simulation used a different decision-support tool: (1) Current standard, (2) Current standard plus a prediction model, (3) A structured data presentation tool, and (4) A structured data presentation tool plus the prediction model. Clinician- and model-estimated risks were compared, the treatment suggestions from each site were compared, questionnaires about user perceptions were conducted after Simulations 2, 3, and 4 using a Google Form link, and a semi-structured interview was conducted at each site after the last simulation.

**Results:**

Similar distributions of risk groups between clinicians and models were found; however, distinct discrepancies in predictions arose, particularly with higher-risk patients, highlighting the need for standardization for more complex clinical cases. The primary perceived benefit of decision support was increased standardization of care, independent of the individual physicians’ personal views. However, participants emphasized the necessity of clinician autonomy to overrule tool suggestions when identifying clinical nuances not captured by the model.

**Conclusions:**

The colorectal cancer MDTs expressed a positive view regarding the use of prediction models and other forms of decision-support in their workflow. While clinicians and prediction models had similar risk score distributions, they diverged in the assessment of specific individual patients.

## Introduction

The multidisciplinary team (MDT) conference has been regarded as a cornerstone of optimal treatment of cancer for over a decade in several countries globally, including Denmark [1]. However, previous studies have not systematically demonstrated that patients discussed in preoperative MDT conferences experienced improved surgical or oncological survival [1–3]. In Denmark, this may be attributed to the presence of national guidelines that group patient in overall categories and tend to be adhered to regardless of whether an MDT discussion is conducted. MDT conferences are resource-intensive, consuming several hours weekly from multiple specialists across various disciplines. Consequently, the lack of evidence for improved care should serve as a catalyst for reconsidering how MDT conferences are conducted [4, 5]. Perhaps they could be more effective if they resulted in a more personalized approach to treatment that fits optimally with each individual patient.

We developed a machine learning (ML) model, AID-SURG [12], to predict 1-year mortality after elective, curatively intended colorectal cancer surgery. This algorithm was used to stratify patients into risk groups, facilitating corresponding individualized prehabilitation [6–11] and perioperative optimization care bundles [12]. AID-SURG resulted in a significant reduction in medical complications, with the highest-risk patients deriving the greatest benefit in terms of reduced complications [12].

A decision-support tool such as the AID-SURG model has not been tested in a multicenter setting. Formal research on decision-support tools, specifically focusing on how data presentation impacts decisions, is also lacking. Furthermore, MDT conferences lack a formal structure regarding the clinical information required for a comprehensive discussion, often yielding insufficient patient presentations and, consequently, suboptimal discussions. To address this, we developed a visual MDT tool named MDT+, which provided a structured MDT format including demographics, clinical variables, comorbidities, cancer topography, and biochemistry, enriched with the AID-SURG model.

It is well recognized that many tools, such as prediction models, are rarely implemented in clinical settings. This can be due to user skepticism, tool being too complex for daily use, models not being trained in a contemporary surgical setting, the use of variables unavailable at the time of the MDT, or an impractical data input interface [13–18]. Understanding perceptions of both the tool design and the use of a prediction model is crucial for future efforts to implement personalized care bundles that can reduce complications and costs [12].

The aim of this multicenter simulation study was to explore user perceptions of four different decision-support modalities, and how these impacted the decision-making process. Additionally, we aimed to investigate the feasibility of using a decision-support tool to facilitate discussions about prehabilitation for patients with colorectal cancer.

## Methods

**Figure 1:**
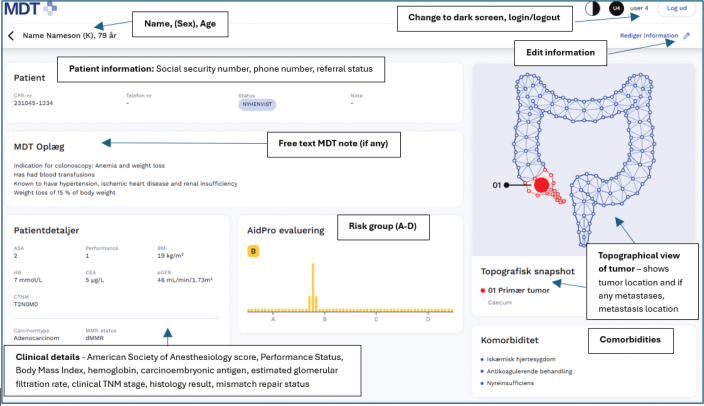
Web application (MDT+ application) used for the simulated multidisciplinary team conference for each study site.

Four hospitals specializing in colorectal cancer surgery participated in four simulated MDT conferences each. None of the hospitals had utilized any decision-support tools as the standard of care in regular MDT conferences. They received information regarding MDT+ and the AID-SURG model prior to the simulations (Figure 1).

The four simulations incorporated varying levels of decision-support as outlined in Table 1. The first simulation utilized only a free-text description of the case (St); the second provided a free-text description of the case combined with the AID-SURG risk group (St + ML); the third utilized the MDT+ application for case presentation (MDT+ only); and the fourth provided a case presentation using MDT+ enriched with the AID-SURG risk group (MDT+ + ML). Each simulation series conducted used a total of 24 patients (6 per simulation). The patients had previously undergone surgery at another colorectal center at least 1 year prior to the study.

**Table 1:** Different multidisciplinary team conference modalities.

	Current standard (St)	AID-SURG model (St + ML)	MDT+ patient overview (MDT+ only)	MDT+ combined with AID-SURG model (MDT+ + ML)
Patient data are presented in free text	+	+ (when used along with standard MDT notes)	+	+
Contains personal data (social security number, age, sex, name, etc.)	+	+ (when used along with standard MDT notes)	+	+
Can be accessed by any smartphone or company laptop	+ (if distributed as Word or PDF)	+	+	+
Contains individual risk predictions to support decisions	–	+	–	+
Has care bundles corresponding to risk assessment	–	+	–	+
Contains a structured data overview, where unique datapoints (such as performance status or laboratory results) are always in the same position	–	–	+	+
Encourages user to fill in clinical data points such as laboratory results	–	–	+	+
Encourages the same conference structure in all hospitals where it is implemented	–	+ (if prehabilitation bundles are implemented)	+	+

AID-SURG: [12]; ML: Machine learning; MDT: multidisciplinary team.

### Study setting

Each site hosted simulated MDT conferences with the presence of an observer from the research team. To ensure a realistic study environment, all simulated MDT conferences were held in the same room used for daily clinical practice, with the attendance of the same representatives from each clinical specialty as consistent with standard practice at each study site. If a specialty was not present due to illness or other engagements, a research team member assumed the role based on information given by a specialist in the respective specialty. This occurred in five out of 16 simulations and only happened with non-clinical specialties. As many clinicians as possible from each of the four sites’ MDT teams were encouraged to participate; multiple clinicians participated in all four simulations across all centers. Teams consisted of a mix of colorectal surgeons, oncologists, pathologists, and radiologists, reflecting normal practice in Denmark and Norway.

### Patients and stratification

**Figure 2:**
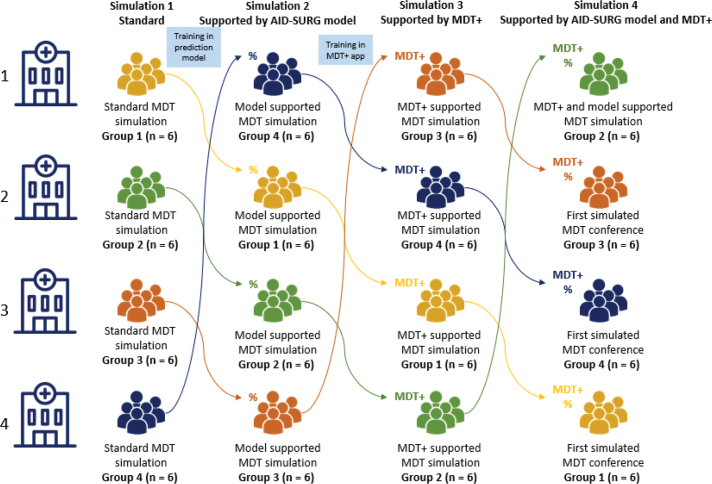
Schematic overview of study steps. Four hospitals were included with six patients per simulation. For each simulation, the patient groups were shuffled to a new hospital to avoid recall bias. The simulations were as follows: (1) standard MDT conference; (2) standard MDT referrals supported by the prediction model; (3) MDT+-supported MDT conference without prediction models; and (4) MDT+-supported MDT conference with prediction models. Depiction of the features of each modality of patient information in the four simulations including: Simulation 1 (current standard) only using a MDT referral note, simulation 2 (standard and prediction model), simulation 3 (the MDT+ interface where there is a structured overview of patient information without the model), and simulation 4 (a combination of the model and MDT+ overview). The prediction model that is used is the AID-SURG prediction model [12]. Below is a presentation of what each modality has (+) and does not provide (-).

The 24 patients were split into four groups of six patients, yielding four conferences with different patients in each simulation (Figure 2). Prior to grouping, the patients’ model-estimated risk of 1-year mortality risks was calculated, and they were stratified into one of four risk groups (A–D): Group A had very low risk (<1% risk), Group B had low risk (1–5% risk), Group C had moderate risk (5–15% risk), and Group D had high risk (>15% risk). In each of the four simulations, there was one patient from Group A, one from Group B, two from Group C, and two from Group D, to facilitate the discussion of patient cases with higher risk and decision complexity. Some of the features of MDT+ and the AID-SURG model can be viewed in Table 1. Prior to each simulation, clinicians were trained in using the decision-support tool and its different scenarios and available adjuncts. All four centers simulated each of the four scenarios, yielding a total of 16 simulations.

### Data collection

Data on decisions were gathered throughout each simulated MDT by the surgeon serving as team leader. Subsequently, questionnaires were administered after each simulation involving a decision-support tool (Simulations 2, 3, and 4). Questions focused on facilitators and barriers to implementing the tool in future MDT conferences. The Modified Clinician’s Guideline Determinants Questionnaire [19] (Supplementary Table 1) was adapted to cover the idea of decision support and to cover the specific support modality from each simulation. Responses to questions were rated on a Likert scale from 1 (completely disagree) to 7 (completely agree); however, a few questions were answered with a single-choice button or a free-text response (Supplementary Table 1). The questionnaire was disseminated using Google Forms.

Finally, after the final simulation, as many team members as possible participated in a focus group interview exploring their experiences with and opinions on using the decision-support tools. The interviews were conducted by the first author, a female surgical resident, and a PhD fellow. The interviewer had participated in multiple colorectal cancer MDT conferences prior to the simulations and was also the facilitator of training sessions and the simulations themselves.

### Analysis

Interview data were largely reported using the Consolidated Criteria for Reporting Qualitative Studies (COREQ) checklist [20]. The checklist is split into three domains: research team and reflexivity, study design, and analysis and findings.

The initial analysis of focus group interview data took an inductive approach [21–23], where the interviewer reads the transcripts and color-coded essential quotes highlighting recurring themes. The thematic analysis was facilitated and supervised by an expert qualitative researcher.

### Statistical considerations

Results obtained from decisions during conferences and questionnaires were analyzed statistically using R v.4.2.0 and the tidyverse package for RStudio. Data were reported descriptively and, when relevant, converted from counts to percentages. Categorical variables, namely, Likert scale values in questionnaires, were reported as medians with interquartile ranges (IQRs) when appropriate.

## Results

A total of 28 clinicians participated across simulations, including 19 specialist colorectal surgeons, three oncologists, three radiologists, two pathologists, and one specialist in clinical physiology and nuclear medicine. The questionnaire respondents included 19 surgeons, two oncologists, two radiologists, and one pathologist. Twelve participants participated in the interviews: 10 surgeons and two oncologists, comprising two women and 10 men, aged 39 to 58 years. Interviews lasted an average of 35 min (range: 26–40 min).

### Decisions

A total of 96 decisions were made for 24 different patient cases. The distribution of decisions can be found in Table 2, and the individual treatment suggestions for each patient are listed in Table 3.

**Table 2:** Distribution of model predicted risk groups compared to the actual predictions made by clinicians throughout the four simulations – a total of 96 decisions were made over the course of the four simulations on the four sites.

	Model prediction (%)	Clinician estimate (%)
Risk group A	17	20
Risk group B	17	17
Risk group C	33	34
Risk group D	33	29

**Table 3:** For each patient, the table displays the treatment suggestion, the risk group (A–D) placed by the MDT-conference with the applicable decision support, and the model-estimated risk group (A–D) (in parentheses).

	Center A	Center B	Center C	Center D
	Standard MDT conference (no decision support)	Standard referral notes and model	MDT+ overview without model	Combination of model and MDT+ overview
Patient 1	Sigmoid resection	Sigmoid resection	Sigmoid resection	Sigmoid resection
	A (A)	A (A)	A (A)	A (A)
Patient 5	Right hemicolectomy	Right hemicolectomy	Right hemicolectomy	Right hemicolectomy
	B (B)	B (B)	B (B)	B (B)
Patient 13	Right hemicolectomy	Right hemicolectomy	Immunotherapy	Immunotherapy
	C (C)	C (C)	C (C)	C (C)
Patient 14	Right hemicolectomy	Right hemicolectomy	Right hemicolectomy possibly with stoma	Right hemicolectomy with terminal ileostomy
	C (C)	C (C)	C (C)	D (C)
Patient 21	Good talk with patient about risks and wishes – if surgery then right hemicolectomy	Right hemicolectomy	Good talk with patient about risks and wishes – if surgery then right hemicolectomy	Good talk with patient about risks and wishes – if surgery then right hemicolectomy
	D (D)	D (D)	D (D)	D (D)
Patient 22	Right hemicolectomy	Right hemicolectomy	Right hemicolectomy	Right hemicolectomy
	D (D)	D (D)	D (D)	D (D)
	Center A	Center B	Center C	Center D
	Combination of model and MDT + overview	Standard MDT conference (no decision support)	Standard referral notes and model	MDT+ overview without model
Patient 2	iAPE		iAPE	PME/iAPE
	A (A)	A (A)	A (A)	A (A)
Patient 6	Right hemicolectomy	Right hemicolectomy	Right hemicolectomy	Right hemicolectomy
	B (B)	C (B)	C (B)	B (B)
Patient 15	Right hemicolectomy	Right hemicolectomy	Immunotherapy	Immunotherapy
	C (C)	C (C)	C (C)	C (C)
Patient 16	Colonoscopy though ascending leg of stoma	Colonoscopy though ascending leg of stoma	Colonoscopy though ascending leg of stoma	Colonoscopy though ascending leg of stoma
	C (C)	C (C)	B (C)	C (C)
Patient 23	New biopsy and if malignant then radiation	TEM as biopsy and if not free margins then radiation	TEM7TEO	TEM and depending on histology radiation
	D (D)	C (D)	C (D)	D (D)
Patient 24	Compromised (palliative) resection or palliative transfusions	Extended right hemicolectomy	Extended right hemicolectomy	Downstaging (or complete) immunotherapy and if not complete response then extended right hemicolectomy
	D (D)	D (D)	C (D)	D (D)
	Center A	Center B	Center C	Center D
	MDT+ overview without model	Combination of model and MDT+ overview	Standard MDT conference (no decision support)	Standard referral notes and model
Patient 3	Rectal resection with anastomosis	Rectal resection with anastomosis	Rectal resection with anastomosis	
	A (A)	A (A)	A (A)	A (A)
Patient 7	Right hemicolectomy	Right hemicolectomy	Right hemicolectomy	Right hemicolectomy
	A (B)	A (B)	B (B)	B (B)
Patient 17	Right hemicolectomy	Right hemicolectomy	Right hemicolectomy	Right hemicolectomy
	C (C)	C (C)	C (C)	C (C)
Patient 18	Downstaging chemo and referral for T4 center	Downstaging chemo and referral for T4 center	Try laparoscopic otherwise open PME with excision of pre-sacral fascia and possibly stoma	Referral for T4 center
	C (C)	C (C)	C (C)	C (C)
Patient 25	Very extended right hemicolectomy	Referral for T4 center	Open subtotal colectomy with anastomosis	Open subtotal colectomy with terminal ileostomy
	C (D)	D (D)	D (D)	C (D)
Patient 26	New colonoscopy, radiation if treatment, otherwise palliative stent and transfusions	Good talk about risks and if patient wants surgery, then APE	Defunctioning stoma and CT-colography	Defunctioning stoma and possibly radiation treatment
	D (D)	C (D)	D (D)	D (D)
	Center A	Center B	Center C	Center D
	Standard referral notes and model	MDT+ overview without model	Combination of model and MDT+ overview	Standard MDT conference (no decision support)
Patient 4	iAPE + MRI of liver	TME with temporary stoma + MRI of liver	iAPE + MRI of liver	TME or iAPE + MRI of liver
	A (A)	A (A)	A (A)	A (A)
Patient 8	Talk to patient – either double segment resection or subtotal colectomy	Left flexure resection and right hemicolectomy	Left hemicolectomy and subsequent colonoscopy	CT colonography + sigmoid resection with anastomosis)
	B (B)	B (B)	B (B)	B (B)
Patient 19	Good talk with patient about either extended right hemicolectomy or palliative care	Extended right hemicolectomy	Optimization with internal medicine specialists and subsequent extended right hemicolectomy	Optimization with internal medicine specialists and subsequent extended right hemicolectomy
	D (C)	C (C)	C (C)	D (C)
Patient 20	Good talk with patient about either extended right hemicolectomy or palliative care	Extended right hemicolectomy	Right hemicolectomy	Optimization with cardiology and subsequent right hemicolectomy
	D (C)	D (C)	C (C)	C (C)
Patient 27	New biopsy and subsequent radiation	New biopsy and subsequent radiation. If responding well to prehabilitation possibly APE	New biopsy and subsequent radiation	New colonoscopy
	D (D)	C (D)	D (D)	D (D)
Patient 28	If candidate for surgery, then sigmoid resection with colostomy	Palliative care	Either sigmoid resection or palliative care	Radiation
	C (D)	D (D)	C (D)	D (D)

MDT: multidisciplinary team; PME: Partial mesorectal excision; iAPE: Intersphinteric abdomino-perineal excision; TEM: Transanal endoscopic microsurgery; APE: Abdomino-perineal excision; MRI: Magnetic resonance imaging; CT: Computed tomography scan.

Green cells indicate agreement between the model and the multidisciplinary team; red cells indicate discrepancy.

### Questionnaires

The 24 clinicians completing the questionnaire all agreed that guidelines support decision-making. All participants had been exposed to some level of decision support during their career, whether via tools like MDT+ or simply national guidelines. When asked about their opinions on perioperative optimization and using a tool to structure prehabilitation, all rated 5 or above on the Likert scale. There was a general agreement that using a tool for stratification for perioperative optimization would improve outcomes and lower complications while giving a sense of better decision confidence.

In contrast, 19 out of 24 clinicians rated the disadvantage of decision support in terms of time and financial costs between 1 and 3 on the Likert scale, indicating they did not view decision support as a potential financial or time burden. Only five clinicians provided a rating of 4 or higher.

In the free-text responses (see the supplementary material for questions), participants generally felt they were sufficiently prepared for simulations following pre-simulation training and found that the support tools often provided predictions that met their own expectations. In this free-text section of the questionnaire, participants were asked what the main reasons they would or would not use the MDT+ tool. The most common answers to why they would use the tool included: (1) ‘Better optimization of fragile patients’, ‘Better allocation of different tools for patients’ and similar variations, (2) ‘Better cross-specialty understanding of operability and risk’ and ‘Less interoperator variability’, (3) ‘Standardization’, and (4) ‘Better patient care’. The most common reasons for not wanting to implement MDT+ was (1) ‘Cost’, (2) ‘Time commitment’, (3) ‘The foundations of the tools are already used in clinical practice (prehabilitation), and (4) ‘No reason not to use it’.

In the Likert scale answers, the consensus revealed a positive impression (score 4–7) of decision support in general as well as for each system provided. Generally, surgeons were positive (score 4–7) about each modality. Regarding layout and usability, MDT+ was rated a median of 6 (IQR: 4–7). In terms of clarity of results, the AID-SURG model alone scored a median of 5 (IQR: 4–6), compared to a median of 6 (IQR: 4–7) when using it in conjunction with MDT+. Regarding the likelihood of daily implementation of MDT+ with AID-SURG, the median score was 6 (IQR: 4–7), and regarding perception of improved clinical discussion, the combined tools scored 6 (IQR: 4–7) on average. Most participants, 67% of the respondents, preferred the combination of the two tools.

### Interviews

The interview analysis initially identified seven recurrent topic areas: clinician autonomy, increased standardization of treatment, the discussion with the patient, identity as physicians, the use of emotions in MDT notes, potential financial benefits and disadvantages, and the use of risk percentages versus risk groups. Through iterative analysis, these were further examined in relation to the study aim, and two overarching implementation-related themes were identified as central to the participants’ accounts: clinician autonomy and increased standardization of treatment.

### Clinician autonomy

It appeared that the surgeons and oncologists treating colorectal cancer patients carry a sincere feeling of responsibility for treatment decisions and outcomes. Consequently, some participants were skeptical that the inclusion of a ML-based tool might impact how patients are treated. Specifically, there was a concern that patients evaluated as high-risk by the model might not be offered surgery due to the predicted high risk, even if they would have been offered surgery under the current standard of care. One participant noted, *‘I can worry whether we will remain critical towards the suggestions made by the model over time. When you work with a system every day, the likelihood of questioning the model may decrease over time’,* while another stated, *‘I think there is a risk of surgical undertreatment because people [the team members] will worry too much’.* Some voiced the importance of being able to disagree, not negatively, but as a general necessity: *‘It is important to me that I can also disagree with the algorithm[model]. Like, so we can reflect over: ‘Do we actually think this is a C-patient, or do we actually think this is something completely different?’’*

An example of this concern regarding the risk of undertreatment involved a patient with a cecal tumor who had continuous bleeding, resulting in low hemoglobin levels and a recurring need for transfusions. Due to several comorbidities, relatively impacted functional capacity, and low hemoglobin levels, the patient was placed in risk group D. This prompted many team members to debate whether she should be operated on, until one surgeon loudly exclaimed: *‘But she is bleeding!’* Subsequently, most of the team adopted a new stance, considering how removing the tumor might increase both quality of life and the likelihood of survival, as the patient would not be constantly bleeding. In the subsequent interview, the surgeon’s colleagues expressed: *‘When we had the first simulation, and [Name] said ‘But she is bleeding!’, that is the bottom line of it, right? It is all good with the model and such, but it is dangerous to have hemoglobin levels of 4.3 mmol/L. That is after transfusion. Should we postpone the surgery? What are we waiting for?’*

However, many also voiced that they felt in control of the plan regardless of what the model suggested: ‘I keep the freedom to say that despite the algorithm[model], I want to do something different – while knowing that it is a high-risk decision’. Additionally, some considered how collaboration with the model’s suggestions could be improved by increasing the transparency of the factors driving a specific risk: ‘It would be interesting if you somehow could see what drives the risk for the individual patient. Especially because then you would be able to see if it was actually something we could change, such as anemia or a low BMI, or if it is stationary, such as high age, which we must assume is a risk factor’.

Overall, there was a concern that a level of dependency on the tool would develop over time. This could lead to a decreased level of clinical or critical thinking, which is likely why the theme of autonomy to deviate from model suggestions was recurring. However, despite these concerns, many expressed relief at the possibility of more consistent decisions for specific types of patients, which was reflected in the second major theme.

### Increased standardization of treatment

While some participants voiced concerns about over-dependency on the model, all agreed that the main advantage of the MDT+ tool was the expectation that discussions would be less impacted by differences in surgeons’ personal beliefs: *‘We bring our own identities. It is who we are. The information is very biased, and depending on who attends the conference, the decisions may be completely different’.* Some clinicians noted how this information would diminish the emotional element in conferences by making information distribution less biased and more objective. One dialogue illustrated this: *‘P1: I think we all do it very differently; however, us who sit here tend to say the same things to all patients coming through the door. P2: Yes, exactly, but there are those people who are like: ‘Oh it is sad for him, and he has a vacation home in Provence etc…’ P1: Yes, it is all good, [Name], but now you have promised him something that you actually don’t know if you can keep’.* Another participant remarked, *‘I think this system for presentation of patients can add a higher quality in the discussion and remove the emotional component. The emotions are important when we are with the patients, but during conference it is just noise’.*

Additionally, the hope was that this standardization would lead to improved treatment, with preoperative optimization before surgical treatment becoming more streamlined: *‘You have the opportunity to adjust so to say. It is important and then there is also the opportunity to standardize these things. That is the value for me’.* Many expressed this opinion in detail: *‘This system makes for a more streamlined discussion in the MDT, but it will also help to streamline the treatment initiated in the clinic. Currently, we don’t discuss optimization, so it is only the surgeons who are really into that who talk to the patient about it. It will be hard for the ‘old’ surgeons to avoid taking optimization into consideration when there is a conference decision about it’.* Another added, *‘I can mention the people who have the same opinions, I can mention who prescribes prehabilitation too aggressively, and I can mention who prescribes too little, but we can’t throw them out of the conference anyway. But there is another commitment if we have standardized approaches to prehabilitation’.*

One surgeon summarized it concisely: *‘It is actually a nice setup, because we all supplement each other a lot and we cover all bases. I mean it would be a shame if we were all the same, but that doesn’t mean that our decision tool should not be founded on solid data and ensure a certain standard’.*

All participants agreed that standardization was the major advantage, as some hospitals were already considering prehabilitation efforts for their patients but lacked a structure for allocating patients to different interventions: *‘We already do many of these things* – *or we feel like we do* – *but putting things into such a standardized system will help maintain quality throughout the entire conference, even when there are many patients’.* Some even considered how they could use the model to facilitate discussions with patients in the clinic: *‘I think that a specific risk group will make it easier to talk with the patient and explain why we recommend what we recommend. It can be difficult to motivate sick people to exercise, but you can probably have a more logical conversation with the patient* – *also the conversation of whether we should operate at all* – *if you can support it with their own data’.*

Interestingly, many surgeons considered themselves to be aligned with the model, but felt their colleagues were more misaligned: *‘I don’t feel like I am particularly far off the model predictions’,* and *‘Foundationally, we have different approaches to what patients can and when the situation is sad for someone’.*

The considerations about standardization by stratification, the implementation of optimization in the MDT conference discussion, and the prospect of using the decision-support tool in clinical life were overwhelmingly positive.

Overall, across all participating centers, clinicians highlighted several perceived advantages of MDT+, the AID-SURG model, and the combination of the two. The most frequently cited benefit was the potential to standardize prehabilitation and perioperative care. Participants emphasized that such standardization could reduce interoperator variability in MDT decision-making and ensure more consistent preoperative optimization across institutions, ultimately promoting greater equality in patient care. Many clinicians also found it reassuring that the risk prediction model frequently aligned with their own clinical judgment, serving as a useful validation tool rather than as a prescriptive directive. However, there were concerns about overdependence on the model, and the need for autonomy to make decisions differing from the model without repercussions was essential for the surgeons and repeatedly mentioned during interviews. Overall, when asked directly, all participating MDTs found value in the MDT+ application and the approach to multidisciplinary discussion with it, and there was a great interest in possibly adapting the tool in their scope of practice in the future.

## Discussion

In this multicenter simulation study, we did not find a significant shift in the overall distribution of patients across risk groups. Individual-level reclassification did occur; however, many clinical decisions about treatment remained relatively consistent between centers. Additionally, questionnaires and interviews regarding user-perceived strengths and weaknesses showed generally high trust in the MDT+ application and the prediction model, an expectation of less variability in decisions among the MDT, and a positive perception of the tool in general, indicating both the feasibility and clinical relevance of digital decision-support tools.

The increasing complexity of cancer care has increased the importance of MDT discussions in treatment planning to ensure optimal care. However, the quality and consistency of MDT recommendations can be variable, particularly in complex cases of CRC [3, 24, 25]. Recently, there has been increased scientific interest in how to create and use decision-support tools to improve the reproducibility and quality of MDT decisions, especially by reducing inter-clinician variability and facilitating evidence-based practices [26, 27]. MDT+ adds to this by combining predictive modeling with practical care pathways directly integrated into MDT workflows. Importantly, the users of MDT+ emphasized that the tool was easy to navigate, allowed flexibility to integrate clinical experience, and supported rather than overruled clinician judgment. Prior studies have also suggested that decision-support tools must honor the balance between digital automation and clinical autonomy [24, 28–31].

The use of real-life MDT simulations across four clinical centers allowed us to explore not only the technical integration of MDT+ and the AID-SURG model but also the human contributions of discussion between high-level professionals in a specialized clinical setup [32]. Simulation-based evaluations of decision-support tools are increasingly recognized as a vital step prior to full clinical implementation due to a feasible and standardized setting, lower resource requirement compared to full-scale trials, and the ethical advantage of not using an untested tool to impact real patient trajectories [33–35]. This is an effective and patient-safe first step to identify technical issues, training needs, and barriers and facilitators for use and implementation [36].

We found high general acceptance and intuitiveness of MDT+ among participants. Results from the questionnaires given after each simulation showed trust in the algorithmic predictions and openness to modifying decisions based on tool outputs if the clinician always has the final call, in line with current evidence [37]. Historically, there has been a tendency to have difficulty implementing decision-support tools; thus, we were rather surprised to see more positive responses regarding implementing guidelines, decision-support tools, and models in the MDT conference room. There was an expectation that parts of the teams would be more negative toward changing the current MDT practices. However, a reason for this could be that team members who replied to the questionnaires (24 out of 28) might be more positive about the project and the idea of adding decision-support to MDT conferences, which would introduce a degree of selection bias to the results from the questionnaires.

Questionnaire responses said that most users preferred the combination of MDT+ and the AID-SURG model, but during interviews, it seemed that several users found the model the most useful part of the decision-support tools and therefore considered the combination of the two a little more redundant. All in all, the questionnaires revealed that clinicians tended to be positive in terms of working with new tools to support decisions and discussions with both colleagues and patients about treatment; however, there were concerns about possible overdependence on support aids.

Two major themes were identified during interviews through inductive content analysis, and the themes seemed generally slightly opposing. These were concerns about clinician autonomy and appreciation of increased standardization.

This led us to consider two major questions: (1) What does the model provide, that we do not currently have? and (2) When there is a proven effect of model stratification for selection to perioperative optimization, what are the clinicians’ concerns? We found across observations, simulations, and interviews that clinicians consistently argued that the largest advantage of the AID-SURG model was to bring structure to the discussions during MDT conferences and more focus on relevant prehabilitation. Although Denmark has had a national guideline for prehabilitation in colorectal cancer since 2023 [38], the indications for prehabilitation interventions remain relatively vague, and surgeons feel less knowledgeable in areas such as nutrition and physiotherapy. The risk groups and corresponding care bundles offered a sense of objectivity and a common language for decisions in this area. Interview participants often felt the tool aligned with their own risk estimates and could help ensure sufficient perioperative optimization even when colleagues with less focus on this were in charge of treatment.

Clinicians, who tended to see a great value in prehabilitation even before participation in this study, mentioned that prehabilitation decisions are currently made after the MDT conference and due to variation between clinicians, patients may be offered different interventions. They considered that if the MDT conference collectively agreed on a plan supported by the model-estimated risk group, recommendations could be more standardized and limit inter-clinician variation. Additionally, using the prediction by the model to support MDT decisions could enhance and personalize the following conversation with the patient encouraging better compliance with the intensive programs set for frailer patients.

These benefits contrasted with the first theme of maintaining autonomy to deviate from the model predictions based on clinical judgment, which leads to the second question regarding the clinicians’ concerns. The interview participants emphasized concerns that the model might not grasp clinical nuances, such as some risk-increasing traits being non-modifiable by optimization (such as high age or decreased mobility due to leg amputations) or traits related to the tumor. The case of the bleeding patient is a good example of this concern. In that case, the patient was classified as a group D patient among other things due to severe, transfusion-requiring anemia and low performance status, likely as a result of the anemia. The model focused on these values independently, but the team of clinicians could recognize that the reason for her low functional capacity could likely best be mitigated through surgery – a holistic perspective outside the scope of the model. There were a few complex cases like this, where being low-risk or high-risk was not simple and resulted in deeper discussions about whether to stick with the system recommendations. Despite this, several interviewees specifically highlighted that they often agreed with the model, making it clear that complete trust in the model is probably not possible, knowing that it cannot take complex clinical patterns into consideration, but that most cases were simple enough to result in agreement with the clinical assessment and model suggestion.

In addition to the concerns about how complex cases were perceived by the model, participants expressed worry about overdependence on the tool over time. It seems that some of this concern also stemmed from uncertainty about having a digital tool suggesting clinical decisions over skilled surgeons, thus removing some identity or responsibility from the physicians, which has also been seen to be a concern in other studies [39, 40].

A key strength of this study lies in its multicenter, simulation-based design. By simulating real-world MDT conference workflows in four centers, we were able to assess not just performance metrics but also clinician interaction with the tool in varying institutional cultures. This enables broader generalizability compared to single-center usability studies. Another important strength is the combined approach to analysis with both quantitative and qualitative outcomes. Combining qualitative interviews with quantitative questionnaires allows for a more well-rounded understanding of the impact of MDT+. Especially, findings from both modalities overall support the same conclusions that a decision-support tool is a valuable contribution to the clinical work; however, interviews suggest that the detailed patient overview was more a nice-to-have than a need-to-have addition to the prediction model.

Several limitations must be acknowledged. While simulations approximate clinical reality, they cannot fully replicate the time constraints and emotional pressures present in real-life. Decisions made in simulated environments may be more exploratory or speculative, with participants being less critical of the tool’s assessment [41–43].

The sample size, while sufficient for qualitative and thematic saturation, is too small to make conclusions about efficacy or patient outcomes. Future trials will need to assess MDT+ in real-time clinical use in a randomized controlled multicenter design to make definite conclusions. Additionally, we tested different levels of the same decision-support tool: The MDT+ tool. Ideally, testing different decision-support tools against each other may have provided us with more nuance in the interviews and questionnaires. Finally, our model predicts 1-year mortality, which, while clinically relevant, does not account for other essential decision factors such as quality of life. Future iterations of MDT+ may benefit from incorporating multidimensional risk models or patient-reported outcomes to enhance holistic care [44].

It is important to acknowledge the bias in the people who chose to join the study and subsequent interviews. These were generally the clinicians with the most interest in the tool, and who also suggested changes and updates to fit it better into their daily work. Clinicians who did not see the same value in MDT+ might have been less likely to spend time on giving feedback.

In conclusion, we found that changes in available decision support did affect the clinicians’ predictions of patient risks, but that clinicians and model generally were close to agreement in terms of estimated risk group of 1-year postoperative mortality. We found that clinicians generally believe that decision-support applications can improve patient care, and the satisfaction with MDT+ was generally high. Although clinicians considered the implications of the tool for the autonomy of their decisions, they also appreciated the standardization of decisions that the tool facilitates. The vast majority of the participants reported that they could envision themselves incorporating MDT+ into their clinical practice.

## Supplementary Material



## Data Availability

As patient data used for presentation in simulations are real-life patient data with personal identifiable information, these data are not available. Responses to questionnaires and other non-personally identifiable data are available upon reasonable request to the corresponding author.
