# Supplementary material for: User perceptions of machine learning models as decision support for colorectal cancer multidisciplinary team conferences (AID-SIM-2): a qualitative simulation study
Source: Acta Oncol. 2026 Jul 31;65:45754. doi: 10.2340/1651-226X.2026.45754 (PMC13434538; doi:10.2340/1651-226X.2026.45754)
Supplement: Supplementary file 1 [file AO-65-45754-s1.pdf]

Supplementary material has been published as submitted. It has not been copyedited, or typeset by Acta Oncologica

Supplementary figure 1

The AID-SIM-2 adapted Clinician Guideline Determinants Questionnaire[18]

Section 1: Background information	
What is your clinical specialty?	Single choice
I generally believe that guidelines optimize health care offerings and outcomes	Yes/no
**Omitted:** Sex, country	
Section 2: Determinants of guideline use	
Have you ever tried any decision support tools for colorectal cancer outside DCCG guidelines?	Yes/no
How would decision support aid you in your scope of practice?	Single choice
I believe that the idea of using a tool to facilitate perioperative optimization makes sense	Likert scale 1-7
I believe that a tool to stratify patients for perioperative optimization would result in a better patient trajectory	Likert scale 1-7
I believe that a tool to stratify patients for perioperative optimization would result in better patient outcomes (e.g. fewer complications)	Likert scale 1-7
I believe that a tool to stratify patients for perioperative optimization would give me better decision competency (support my decisions)	Likert scale 1-7
I believe that a tool to stratify patients for perioperative optimization would contribute with negative side effects in my organization (e.g. time consuming, expensive, etc.)	Likert scale 1-7

I feel sufficiently trained in the mindset behind stratification for perioperative optimization to use a decision-support tool optimally (Knowledge and skill domain put together in one domain)	Likert scale 1-7
I feel that I understand how AID-SURG/MDT+ works sufficiently to use the systems	Likert scale 1-7
I feel that I have the autonomy to deviate from models when my clinical gut feeling suggests this, and I feel that I can argue sufficiently for this decision (such as when we deviate from DCCG guidelines)	Likert scale 1-7
I have confidence in my patients would understand the recommendations of perioperative optimization and would follow their allocated care bundle	Likert scale 1-7
I think that AID-SURG/MDT+ would be easy to incorporate in my current life and possibly in the out-patient clinic	Likert scale 1-7
I think the layout in MDT+ is easy to understand and navigate	Likert scale 1-7
I think the result of the AID-SURG suggestion is crystal clear and not possible to misunderstand	Likert scale 1-7
I generally think that applications and digital tools can help me in my current work	Likert scale 1-7
I think MDT+ fits into out conferences in a time efficient manner	Likert scale 1-7
I think AID-SURG/MDT+ facilitates an improved clinical discussion	Likert scale 1-7
**Added:** The predictions I have seen from AID-SURG/MDT+ correlates to what I expected	Likert scale 1-7
**Omitted:** Professional obligation, normative use by colleagues, expectation of others, organizational capacity for change, guideline tools, underlying evidence, patient preferences	Likert scale 1-7
Section 3: Other determinants not mentioned	

What is the most important reason you would want to use AID-SURG/MDT+ in your current work?	Open ended (free-text)
What is the most important reason you would NOT want to use AID-SURG/MDT+ in your current work?	Open ended (free-text)
Section 4: Learning style	
All omitted	
Additional feedback	
Comments	Open ended (free-text)
